# Root canal debridement efficacy of heated sodium hypochlorite in conjunction with passive ultrasonic agitation: An ex vivo study

**DOI:** 10.34172/joddd.2020.040

**Published:** 2020-10-24

**Authors:** Yogesh Damade, Ramchandra Kabir, Sunanda Gaddalay, Sharvaree Deshpande, Sonali Gite, Sandip Bambale, Nileshkumar Dubey

**Affiliations:** ^1^Department of Conservative Dentistry & Endodontics, Maharashtra Institute of Dental Sciences & Research Dental College, Latur, India; ^2^Department of Cariology, Restorative Sciences and Endodontics, School of Dentistry, University of Michigan, Ann Arbor, MI, USA

**Keywords:** Root canal, Irrigation, Intracanal heating, Sodium hypochlorite, Ultrasonic agitation

## Abstract

**Background.** This study aimed to investigate the endodontic debridement efficacy of different sodium hypochlorite (NaOCl) irrigation regimens with and without ultrasonic agitation, followed by ethylenediaminetetraacetic acid (EDTA) via scanning electron microscopy (SEM) after using a rotary instrumentation system.

**Methods.** Mandibular premolars (n=50) were randomly divided into five experimental groups (n=10) for root canal instrumentation with ProTaper Universal rotary system up to F3. The root canal system was treated with intracanal-heated NaOCl (100°C) or preheated NaOCl (55°C), followed by ultrasonic agitation and EDTA treatment. Samples irrigated with conventional needle irrigation (CNI) using normal saline solution were used as controls. Debridement efficacy was analyzed by SEM. A five-point scale was used to estimate the presence/absence of debris for each canal segment (coronal, middle, and apical). The results were analyzed using one-way ANOVA and post hoc Tukey tests (*P*<0.05).

**Results.** The experimental groups exhibited less debris compared to CNI with saline (*P*<0.05). The amount of debris decreased significantly for the group with NaOCl intracanal heating compared to extraoral heating. Ultrasonic agitation further enhanced the root canal debridement efficacy of NaOCl.

**Conclusion.** In summary, intracanal heating of NaOCl with and without ultrasonic agitation followed by EDTA appears to be a promising method to flush debris from the root canal system.

## Introduction

Clinically, the success of root canal therapy’s success relies heavily on the chemomechanical instrumentation and disinfection of the root canal system.^1^ However, debris produced during mechanical instrumentation penetrates the dentinal tubules and remains adherent to root canal walls, inhibiting intracanal medicaments’ infiltration.^2^ The entrapped debris can act as a potential source of secondary infection, leading to treatment failure.^3,4^ Conventional needle irrigation (CNI) plays a crucial role in eliminating this debris. CNI is not, however, fully effective in delivering irrigants into the intricate areas of the root canal, such as the apical third, dentinal tubules, isthmus, and lateral canals, nor are they universally accepted.^5^ This is because the extent of flushing activity achieved by the CNI technique is only 0‒2 mm from the needle tip depending on the depth of placement and the diameter of the needle, and canal cross-sectional shape and diameter.^5,6^

Research firmly supports that sodium hypochlorite (NaOCl) solution is an ideal endodontic irrigant to flush debris from the root canal system.^5^ Various methods have been studied to increase the potency of NaOCl and enable the use of low concentrations of NaOCl with decreased risk for toxicity or side effects.^7,8^ For example, NaOCl heating enhances the disinfecting and debridement properties due to an increase in the irrigation flow and reaction rate.^9,10^ Increases in irrigant temperatures are accomplished by preheating the irrigant extraorally or heating the NaOCl within the canal by utilizing ultrasonic equipment,^8^ or lasers.^7,9^ The heating of NaOCl often decreases its viscosity, enabling greater penetration, dissolving, and disinfection properties.^10^ Iandolo et al^11^ showed that NaOCl intracanal heating at 180°C significantly decreased debris compared to extraoral heating at 50°C. Besides, the ultrasonic agitation of intracanal-heated NaOCl resulted in improved debris removal relative to CNI and passive ultrasonic irrigation.

Similarly, other studies have evaluated the efficacy of intracanal heating of NaOCl.^12-14^ However, the heating of NaOCl solution above boiling point can cause heat transfer through the dentin, jeopardizing the surrounding tissues, alveolar bone, and periodontal ligament.^15,16^ Very few studies have evaluated the efficacy of NaOCl at its boiling temperature (96‒120°C).^13^ Therefore, this study aimed to evaluate the efficacy of NaOCl at a lower temperature (100°C), thereby reducing the above-mentioned risk and increasing the NaOCl efficacy. Furthermore, removing the debris from the root canal is more significant when EDTA and NaOCl are used in conjunction rather than alone.^17^ Limited data are available on the ultrasonic agitation of intracanal heating of NaOCl solution, followed by EDTA cleaning. Thus, we compared the root canal debridement efficacy of heated NaOCl with and without ultrasonic agitation, followed by EDTA. The null hypotheses were that extraoral heating of this irrigant does not significantly reduce the amount of debris compared with intracanal heating and that there is a significant difference between the percentage of remaining debris with and without ultrasonic agitation.

## Methods

### Preparation of the samples

Fifty mandibular premolars extracted for periodontal or orthodontic purposes were collected at the Maharashtra Institute of Dental Sciences and Research Centre with the donors’ informed consent and Ethics Committee approval (Ref: MUHS/PH-T/E-1/2593/2019). The teeth with root resorption or open apex were excluded. The root segment was obtained by separating the crown of each tooth at the level of the cementoenamel junction. Two longitudinal grooves on the lingual or buccal surfaces of each root were created using a high-speed handpiece with a diamond bur to facilitate the vertical separation of the root segments.

All the root canal instrumentation procedures were performed by a single trained operator. Root canal instrumentation was carried out with the ProTaper Universal system (Dentsply. Maillifer, Switzerland) in the sequence S1-S2-F1-F2-F3, at 200 rpm, and 3 N/cm torque. The root canal was irrigated during the procedure with 5 mL of 5% NaOCl. Intracanal heating of NaOCl was carried out at 100°C using Calamus Dual (Dentsply Maillefer, Ballaigues, Switzerland) with 30/04 carrier tips mounted 3 mm above the working length. Extraoral heating of NaOCl was carried out at 55°C using a kettle. The temperature was controlled using a thermometer. Ten activation cycles, each of 5 seconds, followed by 10 seconds of rest, were performed. After each activation cycle, the NaOCl was renewed, and the files were cleaned to remove debris to maintain effectiveness. The ultrasonic agitation was carried out using an ultrasonic activator (Ultra X, Orikam Healthcare, India). For control, CNI was carried out using normal saline solution with a 30-gauge side-vented needle. The five different protocols of agitation were as follows:

*Group A:* Intracanal heating of 5% NaOCl to 100°C with ultrasonic agitation, followed by EDTA.

*Group B:* Intracanal heating of 5% NaOCl to 100°C without ultrasonic agitation, followed by EDTA.

*Group C:* Extraoral heating of 5% NaOCl to 55°C with ultrasonic agitation, followed by EDTA.

*Group D:* Extraoral heating of 5% NaOCl to 55°C without ultrasonic agitation, followed by EDTA.

*Group E:* Normal saline solution.

### Scanning electron microscopy (SEM)

**Figure 1:**
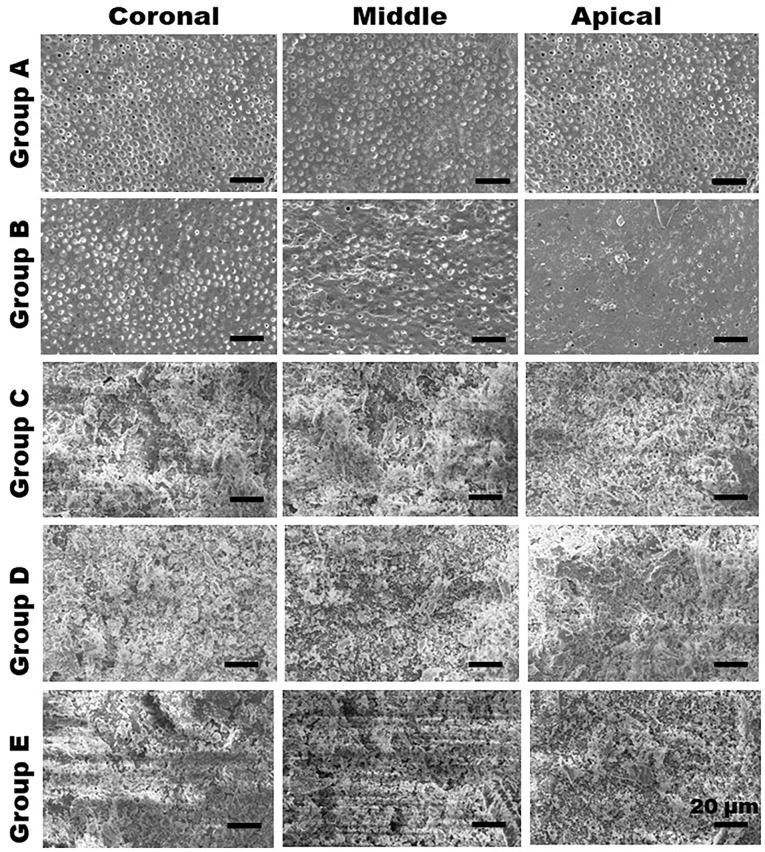
Representative samples of scanning electron microscope images of the root canal dentin surface after final irrigation with extraoral- and intracanal-heated sodium hypochlorite solution with and without ultrasonic agitation.

After root canal instrumentation, a stainless steel chisel was used to split the samples for scanning electron microscopy (SEM, Oxford Incax-act, Carl Zeiss, India) imaging. Briefly, the samples were dried overnight, sputter-coated with gold (EmitechK550X, Emitech Ltd, England), and photomicrographs (Figure 1) in three different areas (at coronal, middle, and apical thirds of the root canal) to examine the presence/absence of debris. Two observers were trained to score the images using the rating system proposed by Hülsmann and Rümmelin.^18^

Hulsmann’s scoring criteria for debris removal:

- Score 1: Clean root canal walls, with or without small debris particles
- Score 2: Few debris agglomerations
- Score 3: Debris agglomerations covering <50% of the root canal walls
- Score 4: Debris covering >50% of the root canal walls
- Score 5: Debris covering the complete or nearly complete root canal walls

### Statistical analysis

Statistical analyses were performed with SPSS (α=0.05, SPSS V. 22, IBM, USA), and descriptive statistics were calculated for mean scores and minimum and maximum scores. One-way ANOVA and post hoc tests were used to compare the mean differences between individual groups.

## Results

**Figure 2:**
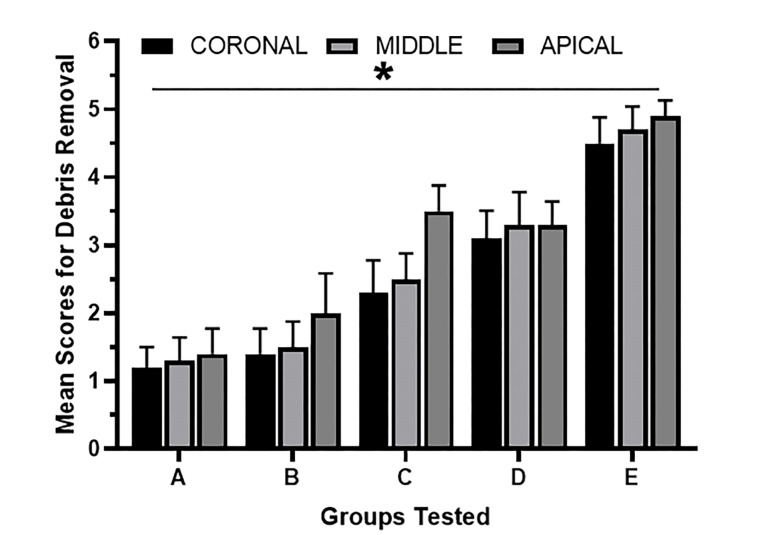
Mean scores of the debris for the coronal, middle, and apical thirds of the root canals for different treatment groups. *Indicates that the difference in the mean is significant at 0.001 compared to control (normal saline irrigation).

The comparisons of debris removal effects and mean value scores of the five debridement regimes are presented in Figures 1 and 2. In groups A and B, dentinal tubule orifices were visible, indicating maximal debris removal from the root canal. On the other hand, groups C and D showed dentin chips and loose particles covering the root canal wall. Specimens irrigated with saline solution (group E) revealed the gross existence of debris completely covering the root canal wall; moreover, debris was packed and adhered into the dentinal tubules. Hulsmann’s score for intracanal heating (groups A and B) of NaOCl, followed by EDTA, revealed a clean root canal with a high score of 1 and 2, with no significant difference throughout the length of the tooth (coronal, middle, and apical thirds). Evaluation of groups C and D showed many agglomerates of debris attached to the root canal wall; group E (control) showed the maximum amount of debris and almost the same amount in all the root canal system portions.

## Discussion

The efficacy of NaOCl depends on the mechanical flush operation and chemical dissolving ability of the solution.^5^ The effect of temperature on enhancing the efficacy of NaOCl has been documented in numerous studies.^10,11,15^ Preheating of NaOCl solution from 22°C to 45°C is one simple way to enhance its debris dissolution ability and antibacterial action. However, the NaOCl will be rapidly buffered in the root canal, minimizing its benefits.^8,11^ Previous studies have shown that intracanal heating of NaOCl solution substantially increases root canal debridement compared to preheated and not heated NaOCl solution.^8,11^ However, there are major differences in the literature regarding the debridement efficacy of NaOCl solution, arising from the use of various experimental conditions and mitigating variables that have affected the outcomes of some in vitro and ex vivo studies.^5,13,19^ As the NaOCl solution boils at temperatures between 96°C and 120°C, it is futile to use the heat carrier above the boiling point.^20^ In the present study, the 100°C intracanal heating of the root canal was efficient for successful irrigation and cleaning the endodontic space. The lower temperature used in this study can potentially prevent the surrounding periodontal complex,^3^ thereby providing safe and effective root canal debridement.

Significant attempts have been made to improve the capacity of NaOCl to remove and dissolve debris through physical fluid agitation using mechanical vibration, ultrasonic agitation, or pulsed lasers.^7,9,10,21^ The typical features of fluid agitation attempts are increasing the fluid’s temperature, enhancing its chemical and biological activity. Presently, “ultrasonic agitation,” which consists of activating irrigants by ultrasonic tips, is the most commonly used technique to enhance NaOCl efficacy.^8,13^ This technique permits the intensified stirring of the irrigant and formation of submicroscopic voids that create shear stress to disrupt debris and damage biofilms physically, thus resulting in a superior cleansing action.^22^ Interestingly, in the present study, there were no significant variations in debris removal between the ultrasonic agitation and no agitation groups with intracanal heating of NaOCl solution, followed by EDTA. These findings are close to those reported in a study by Mayer et al,^21^ showing that NaOCl and EDTA ultrasonic agitation did not reduce the scores of debris in straight root canals compared to the non-activated group.

Nonetheless, this result contradicts many earlier trials where ultrasound agitation led to more successful debris removal than irrigation with a syringe and sonic agitation.^23,24^ These studies are mostly carried out on straight root canals, and various effects can be attributed to disparities in apical preparation, volume, and working times of irrigation and agitation. However, the present study indicated that the use of the EDTA following NaOCl’s intracanal heating resulted in efficient cleaning of the root canal. Thus, the first hypothesis was confirmed: Intracanal heating of NaOCl solution enhanced debris removal. The second hypothesis, however, was not established as there was no significant difference in debris removal over the length of the root canal system, with and without the agitation of NaOCl solution. Although the results are satisfactory, further investigations are required to establish this strategy’s effectiveness in complex root canal anatomies and its impact on the surrounding tissues.

## Conclusion

For complete debridement of the canals, intracanal-heated NaOCl, followed by EDTA, was more effective than extraoral preheating followed by EDTA over the length of the root canal system. NaOCl solution at a temperature of 100°C was as effective as higher temperatures, thereby eliminating the need for heating the irrigants to high temperatures and safeguarding the periodontal complex.

## Authors’ Contributions

YD was responsible for the concept and experimental design, performed the experiments, and wrote the manuscript. RK and SG conceived the idea, hypothesis, and experiment design, and carried out supervision. SD, SG, and SB were responsible for assisting in the experiment and contributed to the discussion. ND was responsible for supervision analysis, interpretation of data, and wring the manuscript. All authors have read and approved the final manuscript.

## Acknowledgments

The authors acknowledge the help of K S Nagaraja Rao from Physics Department, Osmania University, Hyderabad, India for SEM imaging.

## Funding

This research received no external funding.

## Competing Interests

The authors assert no conflicting interests concerning the authorship and/or publishing of this article.

## Ethics Approval

The use of human mandibular premolars and dental pulp stem cells (DPSC) for research was approved by the Institutional Ethics Committee of MIDSR Dental College, Latur (India).

## References

[1] Haapasalo M, Endal U, Zandi H, Coil JM (2005). Eradication of endodontic infection by instrumentation and irrigation solutions. Endod Topics.

[2] Narayan GS, Venkatesan SM, Karumaran CS, Indira R, Ramachandran S, Srinivasan MR (2012). A comparative evaluation on the cleaning and shaping ability of three nickel titanium rotary instruments using computerized tomography-an ex vivo study. Contemp Clin Dent.

[3] Neelakantan P, Devaraj S, Jagannathan N (2016). Histologic assessment of debridement of the root canal isthmus of mandibular molars by irrigant activation techniques ex vivo. J Endod.

[4] Siqueira JF Jr, Pérez AR, Marceliano-Alves MF, Provenzano JC, Silva SG, Pires FR (2018). What happens to unprepared root canal walls: a correlative analysis using micro-computed tomography and histology/scanning electron microscopy. Int Endod J.

[5] Walsh LJ, George R (2017). Activation of alkaline irrigation fluids in endodontics. Materials (Basel).

[6] Munoz HR, Camacho-Cuadra K (2012). In vivo efficacy of three different endodontic irrigation systems for irrigant delivery to working length of mesial canals of mandibular molars. J Endod.

[7] Bago I, Plečko V, Gabrić Pandurić D, Schauperl Z, Baraba A, Anić I (2013). Antimicrobial efficacy of a high-power diode laser, photo-activated disinfection, conventional and sonic activated irrigation during root canal treatment. Int Endod J.

[8] Iandolo A, Abdellatif D, Amato M, Pantaleo G, Blasi A, Franco V (2020). Dentinal tubule penetration and root canal cleanliness following ultrasonic activation of intracanal-heated sodium hypochlorite. Aust Endod J.

[9] Shahriari S, Kasraei S, Roshanaei G, Karkeabadi H, Davanloo H (2017). Efficacy of sodium hypochlorite activated with laser in intracanal smear layer removal: an SEM study. J Lasers Med Sci.

[10] Stojicic S, Zivkovic S, Qian W, Zhang H, Haapasalo M (2010). Tissue dissolution by sodium hypochlorite: effect of concentration, temperature, agitation, and surfactant. J Endod.

[11] Iandolo A, Amato M, Dagna A, Poggio C, Abdellatif D, Franco V (2018). Intracanal heating of sodium hypochlorite: scanning electron microscope evaluation of root canal walls. J Conserv Dent.

[12] Simeone M, Valletta A, Giudice A, Di Lorenzo P, Iandolo A (2015). The activation of irrigation solutions in endodontics: a perfected technique. G Ital Endod.

[13] Iandolo A, Simeone M, Orefice S, Rengo S (2017). 3D cleaning, a perfected technique: thermal profile assessment of heated NaOCl. G Ital Endod.

[14] Amato M, Pantaleo G, Abtellatif D, Blasi A, Gagliani M, Iandolo A (2018). An in vitro evaluation of the degree of pulp tissue dissolution through different root canal irrigation protocols. J Conserv Dent.

[15] Sirtes G, Waltimo T, Schaetzle M, Zehnder M (2005). The effects of temperature on sodium hypochlorite short-term stability, pulp dissolution capacity, and antimicrobial efficacy. J Endod.

[16] Eriksson AR, Albrektsson T (1983). Temperature threshold levels for heat-induced bone tissue injury: a vital-microscopic study in the rabbit. J Prosthet Dent.

[17] Wang H-H, Sanabria-Liviac D, Sleiman P, Dorn SO, Jaramillo DE (2017). Smear layer and debris removal from dentinal tubules using different irrigation protocols: scanning electron microscopic evaluation, an in vitro study. Evidence-Based Endodontics.

[18] Hülsmann M, Rümmelin C, Schäfers F (1997). Root canal cleanliness after preparation with different endodontic handpieces and hand instruments: a comparative SEM investigation. J Endod.

[19] Gopikrishna V, Ashok P, Kumar AP, Narayanan LL (2014). Influence of temperature and concentration on the dynamic viscosity of sodium hypochlorite in comparison with 17% EDTA and 2% chlorhexidine gluconate: an in vitro study. J Conserv Dent.

[20] Woodmansey KF (2005). Intracanal heating of sodium hypochlorite solution: an improved endodontic irrigation technique. Dent Today.

[21] Mayer BE, Peters OA, Barbakow F (2002). Effects of rotary instruments and ultrasonic irrigation on debris and smear layer scores: a scanning electron microscopic study. Int Endod J.

[22] Liang YH, Jiang LM, Jiang L, Chen XB, Liu YY, Tian FC (2013). Radiographic healing after a root canal treatment performed in single-rooted teeth with and without ultrasonic activation of the irrigant: a randomized controlled trial. J Endod.

[23] Salman MI, Baumann MA, Hellmich M, Roggendorf MJ, Termaat S (2010). SEM evaluation of root canal debridement with Sonicare CanalBrush irrigation. Int Endod J.

[24] Jiang LM, Verhaagen B, Versluis M, van der Sluis LW (2010). Evaluation of a sonic device designed to activate irrigant in the root canal. J Endod.

